# Analyzing the process of achieving common wealth for different groups in China based on the opportunity advantage perspective of income distribution

**DOI:** 10.1371/journal.pone.0302876

**Published:** 2024-05-09

**Authors:** Jing Ruan, Xingyu Wang

**Affiliations:** 1 School of Statistics, Capital University of Economics and Business, Beijing, China; 2 School of Data Science, Capital University of Economics and Business, Beijing, China; Alexandru Ioan Cuza University: Universitatea Alexandru Ioan Cuza, ROMANIA

## Abstract

Realizing the common wealth of all people is the essential requirement of socialism with Chinese characteristics. Measuring the process of realizing common wealth and the differences between groups is one of the important issues that need to be addressed urgently. In order to reasonably measure the process of realizing common wealth in China, on the premise of horizontal comparability and vertical consistency, the principles of comparability and consistency are introduced, and a comparative method of opportunity advantage based on income distribution is proposed from the perspective of opportunity equity. Using the 2012–2020 CFPS data to measure and test the opportunity advantages and their differences across regions and groups in China. The study found, firstly, that the opportunity advantage persists but tends to diminish across groups, with the more educated group having a more pronounced opportunity advantage, but that this advantage is diminishing over time. Secondly, the doctoral degree group has a greater probability of earning higher incomes, followed by the master’s and bachelor’s degree groups, but this opportunity advantage, i.e., the probability of earning higher incomes, is diminishing, i.e., the education dividend is diminishing. Third, the difference in opportunity advantage between urban and rural areas still exists, as evidenced by the greater probability of higher incomes in towns than in rural areas, but this advantage has narrowed further over time, with a clear process of urban-rural integration. Fourthly, in terms of gender, men have a certain opportunity advantage over women, but this difference is not significant. Fifthly, in the context of education levels, gender and urban/rural subgroups, under the framework proposed in this paper, China has achieved some success in the process of realizing the common wealth, and is showing a steady upward trend.

## I. Introduction

The report of the Twentieth Party Congress lists "realizing the common prosperity of all people" as one of the essential requirements of Chinese-style modernization. At the same time, "encouraging hard work to become rich, promoting fairness of opportunity, increasing the income of low-income earners, expanding the middle-income group, regulating the order of income distribution, and regulating the mechanism for accumulating wealth" has been placed in a prominent position. Therefore, the path to the realization of common prosperity should emphasize both the promotion of equity of opportunity and the ability to increase the income of the population. Since China’s comprehensive victory in the war against poverty, a large body of literature on the theme of "common wealth" has emerged in the academic community. These literatures mainly contain the connotation, measurement method and realization path of common wealth (Liu et al. [1]; Li, [2]). However, there is little literature to measure the degree of realization of common wealth from the perspective of opportunity equity under the micro perspective. To fill this gap, this paper tries to propose the comparative method of opportunity advantage based on income distribution as a way to measure the process of realizing common wealth.

In the previous literature on measuring common wealth, there is an almost unanimous consensus among scholars that the measurement of common wealth should focus on "common" (or shared) and "affluence" (Li, and Zhu, [3]; Lv and Chen, [4]). While "affluence" is naturally highly correlated with economic development or the income level of the population, "common" or "shared" emphasizes equality of opportunity, especially economic opportunity. Although the interpretation of common wealth should not be confined to the material level, but how to measure the spiritual level is more difficult to grasp, and in the actual life of the residents, in order to make the residents have a sense of obtaining, a sense of happiness, etc., but also depends on the material level, the satisfaction of the spiritual life is mostly based on the material abundance. Therefore, this paper, like the rest of the literature, focuses on the income of the population and the equalization of economic opportunities to increase the level of income.

In recent years, the issue of equal opportunity has received increasing attention from scholars, especially the issue of equal opportunity and social mobility. China’s great project of common prosperity is in fact also a discussion of social mobility, especially the issue of jumping up the income levels of low-income groups. Using parameter estimation and machine learning methods, Shi et al. [5] analyzed how inequality of opportunity affects the leapfrogging of low-income groups from the perspective of inequality of opportunity but did not measure the process of achieving common wealth. In numerous other studies measuring common wealth, most of them focus on constructing a common wealth evaluation index system (Chao and Ren, [6]; Li, [2]; Liu et al.,[1]) There is also part of the literature measured by similar data indicators, such as Wan and Chen (2021) reflecting the quantification of development and sharing in terms of national income per capita and the Gini coefficient of disposable income, and Lv and Chen [4] measuring and analyzing the gaps in income levels, income sharing, and basic public services with respect to the year 2035 by drawing on the Human Development Index.

Regardless of whether it is the indicator system approach or the data indicator approach, these studies have emphasized both sharing and development, and there have been some measurements of the extent to which common wealth has been achieved, but they have all focused more on income levels and income gaps, ignoring the issue of equity of opportunity. Therefore, the comparative advantage approach based on income distribution constructed in this paper from an equal opportunity perspective is necessary and timely to fill this gap in the literature.

From equal opportunity comes another term, opportunity advantage, which involves absolute fairness of opportunity (Rawls, [7]). The equity of opportunity advocated by common wealth needs to be viewed in conjunction with China’s distribution system, which encourages hard work to become rich and more work to earn more, resulting in differences in opportunities for residents to earn higher incomes, and comparing this with absolute equity of opportunity, the group with greater opportunities has an opportunity advantage over the group with smaller opportunities. This paper is concerned with economic opportunity, so this opportunity advantage is expressed as the chance (i.e., probability) of obtaining a higher income. In the income distribution, the opportunity advantage can be further understood as the probability that each income group will receive a higher income, i.e. the probability of dominance. In this paper, the probability of domination is used to characterize the opportunity advantage, and the size of the opportunity advantage difference value is used to measure the process of realizing common wealth. With the solid advancement of common wealth, the people’s chances of obtaining higher incomes should become greater and greater, and the opportunity differences based on the income distribution of the same group should gradually diminish, and the opportunity differences among different groups should be in the appropriate range.

Using the new methodology devised, this paper examines the differences between different groups (by gender, by education, by age) and regions of the country in terms of the opportunity advantages of obtaining higher incomes and charts the country’s progress towards common prosperity. The work done in this paper mainly includes three points: first, combining income distribution and opportunity advantage comparison, designing a new method to measure the process of realizing common wealth that satisfies the principles of comparability and consistency; second, empirically measuring the opportunity advantages and their differences among regions and groups in China and considering the degree of the realization of common wealth in China at the present stage; and third, putting forward recommendations to promote the process of realizing common wealth based on the income distribution’s perspective on opportunity advantages. Thirdly, based on the perspective of opportunity advantage in income distribution, it puts forward recommendations to promote the process of realizing common wealth.

The rest of the paper is structured as follows: the second part is the literature review, the third part is the data and modelling, which mainly includes the opportunity advantage of income distribution, the problem of assessing the income distribution of group m as well as the relevant discussions and proofs of the principle of comparability and the principle of consistency; the fourth part is the process of measuring the process of realization of common prosperity based on the opportunity advantage, and the last part is the discussion and conclusion.

Against this background, this paper would like to explore the dynamic measurement of the opportunity advantage of realizing common wealth and the process of promoting common wealth in the country. This paper will try to answer the following questions: how to combine the promotion of the realization of common prosperity with the opportunity advantage to measure the process of promoting the realization of common prosperity? At the regional level, which regions have greater opportunity advantages in achieving common prosperity? What is the magnitude of differences in opportunity advantages between regions? At the group level, which groups have greater opportunity advantages in the process of realizing common prosperity, and what are the differences in opportunity advantages between groups? The contributions of this paper are mainly reflected in the following aspects. (1) Based on income distribution, it proposes a new method to measure the process of realizing common prosperity in China with the opportunity advantage; (2) It introduces the opportunity advantage into the field of promoting the realization of common prosperity and explores the size of the opportunity advantage of each group in each region from the perspectives of different regions and different groups. (3) From the perspective of the opportunity advantage in realizing common prosperity, it puts forward the relevant suggestions of opportunity compensation and education compensation.

## II. Review of the literature

Opportunity advantages are to some extent expressed as differences in opportunities, i.e. inequality. Inequality is becoming increasingly important in development matters, and it is important for two reasons: (1) inequality slows down the rate of poverty reduction; and (2) high levels of inequality can weaken the foundations of growth. In addition, opportunity advantage has been widely used to study development imbalances and poverty governance. For example, poverty governance has a positive impact on the coordinated development of regional economy (Feng L., et al., [8]), and the effect of poverty governance on regional economic development imbalance shows geographical variability and time evolution. From the perspective of unbalanced and insufficient development to understand the road to the realization of common wealth, it is argued that the transformation from relative poverty to common wealth is embodied in unbalanced and insufficient development (Zhang Y.L., [9]); since then, in solving the problem of relative poverty, some scholars have put forward a similar point of view, arguing that the problem of relative poverty and the realization of common wealth are in the same vein, and that they are both manifested in the unbalanced and insufficient development; Regarding the external manifestations of unbalanced and insufficient development, the main points of view have been put forward, such as urban-rural inequality, regional inequality and inter-individual inequality, and the insufficient development of employment, education, medical care and other functions (Zhou G.H., [10]). It can be seen that unbalanced and insufficient development is the main cause of relative poverty and the biggest obstacle to the realization of the common wealth of all people (Wu Z.L., [11]; Mou C.W., [12]), which has already formed a general consensus among scholars. It can be seen that the application of opportunity advantage has been relatively mature, but no scholars have applied it in the field of common wealth research.

Regarding the quantitative method of common wealth, the current research mainly constructs the common wealth index through the indicator system method (Lv G.M., and Chen X.Y. [4]; Li J.C., et al. [13]), as well as using the Gini coefficient, the Terre index and other indicators to measure it (Wan H.Y., et al. [14]; Tong M.H., et al. [15]; Liu P.L., et al. [1]). These methods constructed the indicator system from the content and dimension of common wealth and measured the degree of common wealth realization. The Gini coefficient and the Thiel index are more widely used in them, but the two are mainly used to measure the gap between the rich and the poor (Zhu Z.C., et al., [16]), which emphasizes on inequality and solves the problem of commonality but does not cover the degree of affluence. Different common wealth indices will differ in their construction process due to the differences in the selected index system, assignment method, synthesis method, etc., which ultimately lead to more significant differences in the empirical results. Therefore, proposing a measurement method to meet the requirements of normativity and universality to measure the process of realizing common prosperity is one of the important problems that need to be solved urgently.

Although the common wealth includes both spiritual and material wealth (Fu C.W., and Gao W., [17]; Li H.J., et al., [18]), the primary problem to be solved in realizing the common wealth is the problem of unbalanced and insufficient development. Spiritual affluence is based on material affluence (Chen Y.J., et al., [19]), and any pursuit of spiritual pleasure cannot be separated from the important constraint of income, and the increase of absolute income level will largely improve the happiness of residents (Luo B.L., and Hong W.J., et al., [20]), and the inequality of income will affect the process of realizing the common affluence. In the study of inequality and poverty governance, the most used and critical indicator is income, which is also often used to measure the economic gap between urban and rural areas, regions, etc. (Cheng C., et al. [21]; Su D.W., et al. [22]; Li C., et al. [23]). The core issue of common wealth is to narrow the income gap, expand the middle-income group, increase the income of low-income earners, and reduce the problem of income inequality between regions and groups (Li J.C., et al. [13]; Hou X.D., et al. [24]; Li S.,[3]), i.e., the process of a more rationalized income distribution.

The connotation of common wealth is not only the pursuit of efficiency, but also the promotion of equity, and scholars generally agree that the two most important elements on the road to common wealth are distribution and development. Some scholars believe that both hands must be grasped and both hands must be hard (Sun Y., and Zhang J., [25]), and some scholars believe that development is the key and distribution is the means (Han L., et al. [26]; Lai D.S., [27]), although the conclusions of the research are different, but none of them has been detached from the core issue of income distribution, i.e., the measurement of the process of the realization of the common wealth cannot be detached from the developmental indicators, but the empty talk about fairness. Although the conclusions of these studies are different, none of them has detached from the core issue of income distribution, i.e. the process of realizing common wealth cannot be measured in isolation from the developmental indicators, and the process of realizing common wealth can only be promoted in the context of a high degree of compatibility and simultaneous development of efficiency and equity. Whereas "equity" often refers specifically to equity of opportunity, highly related to equity of opportunity is opportunity advantage or opportunity difference, which is expressed to some extent as opportunity difference, and is commonly used to study gender differences, urban-rural differences, racial discrimination, income inequality and educational inequality (Yue J.L., et al. [28]; Huang Y., et al. [29]; Li H.Y., et al. [30]; Hou Y.B., et al. [31]). The more pronounced the opportunity advantage, the more inequality is indicated, and the consequence is an increase in income disparity, which deviates from the core assertion of achieving common wealth. Therefore, this paper argues that with the continuous advancement of the great goal of common wealth, the opportunity advantage of gender, urban and rural areas, and different education groups in obtaining higher income should gradually weaken, i.e., the opportunity difference should gradually decrease, which is highly consistent with the important assertion of "promoting fairness of opportunity" made by the Twentieth National Congress of the Communist Party of China (CPC). Therefore, to achieve common prosperity, differences in opportunities must be reduced.

The comparative method of opportunity advantage is based on Rawls’ theory of absolute fairness ([7]), under the assumption of absolute fairness, the opportunities for each group to obtain wealth and status are the same, such as half of the opportunities for men and half of the opportunities for women to obtain the same income (Yao D.Z., [32]). In the process of realizing common wealth, both equity of opportunity and the degree of affluence are the core issues to be measured, so the process of realizing common wealth should be manifested in the fact that the overall opportunity advantage of each group in obtaining higher incomes still exists, but the difference in opportunity between different groups is on a decreasing trend.

Regarding the measurement method of common wealth, the previous literature mostly reflects the index construction method from the macro, but in fact, the measurement of common wealth not only needs to be considered from the macro, but also needs to be measured from the perspective of micro-individuals and meso-groups. In order to make up for the shortcomings of the existing literature, this paper focuses on the degree of common wealth of micro and group. In addition, there is almost a consensus among scholars as far as the existing literature on measuring common wealth is concerned. That is, the measurement method of common wealth is inseparable from the economic level on the macro level and the income level on the micro level, this paper combines the connotation of common wealth, and believes that on the micro and group level, the common wealth is also related to whether the people have equal opportunities to obtain income, and therefore proposes a comparative method of opportunity advantage based on the distribution of income.

To sum up, the opportunity advantage can be explored from the perspective of "equity" to see whether the equity of opportunity has been effectively improved, and it can also be linked with "income distribution" to explore the trend of the probability of obtaining a higher income in order to reflect the "efficiency" of the market economy, thus fully encompassing "efficiency" and "equity", while the differences in opportunity advantage among groups can be used to measure the process of realizing common wealth. In addition, it can be linked with "income distribution" to explore the trend of the probability of obtaining higher income to reflect the "efficiency" of the market economy, so as to fully encompass "efficiency" and "equity", while the differences in the opportunity advantage among groups can be used to measure the process of realizing the common wealth. This paper combines income distribution, opportunity advantage and common wealth, proposes a method of comparing opportunity advantage based on income distribution, and uses it to measure the process of realizing common wealth, with a view to filling the gap in the measurement of the process of realizing common wealth.

## III. Research and data methodology

As shown in the introduction, a comparison of the opportunity advantage of different groups in different regions in the process of achieving shared prosperity can be measured by the probability of dominance. For example, if region A has a better probability of dominance than region B in achieving common prosperity, we assume that region A has a higher probability of entering a higher level of common prosperity than region B. The same is true for inter-group comparisons. In this paper, we translate this probability of having a greater chance of achieving shared prosperity into mathematical terms in a precise assessment formula called the opportunity advantage of achieving shared prosperity. This formula is derived from two intuitive properties, namely the principle of comparability and the principle of consistency.

### (i) Opportunity advantage of income distribution

Constructing a set *M* = {1, 2, ⋯, *m*}, it is containing the m groups. For arbitrary *k* ∈ *M*, all have *f*_*k*_, it represents the probability density function of the *k* population. For a population of m then there is the vector ${\left(f_{i}\right)}_{i=1}^{m}=\left\{f_{i},\cdots ,f_{m}\right\},\left(i\in M\right)$. Take *y* ∈ *R*^+^ to denote the income level of a group. Then *f*_*k*_ (*y*) denotes the number of people in group k whose income is y. There should be $\int_{0}^{+\infty }f_{k}\left(y\right)dy=1$, in other words, all social members of the k-group have been included in the examination.

Without loss of generality, we first consider the case where there are and have been only two groups. Here we discuss the case where there are only two groups, group k and group j. Define the probability of dominance of group k over group j, denoted *q*_*kj*_. This probability of dominance is the most critical factor in assessing the distribution of income between the two groups, and it represents the probability that an individual from group k has a greater chance of earning a higher income than an individual from group j. We call this probability the probability of dominance of k over j, and vice versa. In this paper, this probability is approximated by the frequency of individuals from group k earning more than those from group j. It is important to note that we make sure that respondents from both groups are randomly selected during the study.

To calculate these probabilities, it is more convenient to consider the cumulative distribution function for any probability density *f*_*k*_. The cumulative distribution function is shown by the following formula: $F_{k}\left(y\right)=\int_{0}^{y}f_{k}\left(t\right)dt$, then 1 − *F*_*k*_ (*y*) denotes the probability that a randomly drawn sample from *f*_*k*_ has income above y, denoted by *f*_*j*_ (*y*) the probability that sample *f*_*j*_ has income y, then the probability of dominance of population k over population j, *q*_*kj*_, can be expressed as formula (1):

$$q_{kj}=\int_{0}^{+\infty }f_{j}\left(y\right)\left[1-F_{K}\left(y\right)\right]dy=1-\int_{0}^{+\infty }f_{j}\left(y\right)F_{k}\left(y\right)dy$$ (1)

Similarly, $q_{jk}=1-\int_{0}^{+\infty }f_{k}\left(y\right)F_{j}\left(y\right)dy$, here consider that there are and only two groups *k*, *j*, so we have *q*_*kj*_ + *q*_*jk*_ = 1, so the probability of domination of *k* over *j* and the probability of domination of *j* over *k* can be collapsed as formula (2):

$$q_{kj}=\int_{0}^{+\infty }f_{k}\left(y\right)F_{j}\left(y\right)dy;q_{jk}=\int_{0}^{+\infty }f_{j}\left(y\right)F_{k}\left(y\right)dy$$ (2)

If the probability of domination of *k* over *j* is greater than the probability of domination of *j* over *k*, then group *k* is considered to have an opportunity advantage over group *j*.

### (ii) Assessment of income distribution of m-clusters

For example, China can be divided into 4 regions according to East, Central, West and Northeast, and 8 groups according to education level: doctor, master, bachelor, college, high school, junior high school, primary school, illiterate and semi-literate. Therefore, the number of groups corresponding to the different study subjects is naturally different. To make this study generalizable, we extend it to the *m* > 2 group income distribution for discussion. Firstly, define the m-dimensional density vector $p=\left\{{\left(f_{i}\right)}_{i=1}^{m},\forall \;i\in N,m\geq 2\right\}$, and for any ${\left(f_{i}\right)}_{i=1}^{m}\in p$, define the evaluation function *φ* to be a mapping of *p*, denoted as $\varphi \left({\left(f_{i}\right)}_{i=1}^{m}\right)\in R_{m}^{+}$. Our objective is to determine an evaluation function as a reasonable measure of the opportunity advantage of the income distribution, i.e., the relative likelihood of obtaining a higher income.

Referring to Carmen et al. ([33]), this paper defines an evaluation function in the problem family when and only when it satisfies the principles of consistency and proportionality, then for any *m* ≥ 2, there is a corresponding income distribution whose value can be given by Eq (3).


$$\varphi_{k}\left({\left(f_{i}\right)}_{i=1}^{m}\right)\sum_{j\neq k}q_{kj}=\sum_{j\neq k}q_{kj}\varphi_{j}{\left(f_{i}\right)}_{i=1}^{m}\left(k,j=1,2,\cdots ,m\right)$$ (3)

The applicability and accuracy of this evaluation function is then demonstrated.

First prove that any evaluation function satisfying proportionality and consistency corresponds to the formula that ensures ${\left(f_{i}\right)}_{i=1}^{m}\in P$, for all $\varphi_{1}\left({\left(f_{1}\right)}_{i=1}^{m},\varphi_{2}\left({\left(f_{2}\right)}_{i=1}^{m},\cdots ,\varphi_{m}\left({\left(f_{1}\right)}_{i=1}^{m}\right)\right)\right)$ these values exist, and this proof process is done in two steps.

It is stipulated that *φ* is an assessment function that satisfies the principles of comparability and consistency. For the problem (*f*_1_, *f*_2_), which contains two income distributions, the principle of proportionality determines the expected outcome, so it is not repeated here. Second, discuss the case ${\left(f_{i}\right)}_{i=1}^{m}$, where the probability density (*f*_*k*_, *f*_−*k*_) of the income distribution associated with it is considered for any k when *m* > 2. In accordance with the principle of comparability, i.e., Eq (4):

$$\frac{\varphi_{k}\left(f_{k},f_{-k}\right)}{\varphi_{-k}\left(f_{k},f_{-k}\right)}=\frac{\sum_{j\neq i}q_{kj}}{\sum_{i\neq j}q_{jk}}$$ (4)

Following the principle of consistency and stating that the previous definition of overall dominance also needs to be used here, it follows that.

$$\frac{\varphi_{k}\left({\left(f_{i}\right)}_{i=1}^{m}\right)}{\varphi_{k}\left(f_{k},f_{-k}\right)}=\frac{A_{k}\left({\left(f_{i}\right)}_{i=1}^{m},\varphi \right)}{A_{k}\left(\left(f_{k},f_{-k}\right),\varphi \right)}\to \varphi_{k}\left({\left(f_{i}\right)}_{i=1}^{m}\right)=\varphi_{k}\left(f_{k},f_{-k}\right)\frac{\sum_{j\neq k}q_{kj}\varphi_{j}\left({\left(f_{i}\right)}_{i=1}^{m}\right)}{\sum_{j\neq k}q_{kj}\varphi_{k}\left(f_{k},f_{-k}\right)}$$ (5)

where $A_{k}\left({\left(f_{i}\right)}_{i=1}^{m},\varphi \right)$ denotes the overall advantage of group *k* within the social equity line, while *A*_*k*_((*f*_*k*_, *f*_−*k*_), *φ*) denotes the overall advantage of all other groups except group k. The formula for calculating the overall advantage is presented in Eq (16) below, and without expanding it here, Eq (5) can be collapsed into Eq (6).


$$\varphi_{k}\left({\left(f_{i}\right)}_{i=1}^{m}\right)\sum_{j\neq k}q_{kj}=\sum_{j\neq k}q_{kj}\varphi_{j}{\left(f_{i}\right)}_{i=1}^{m}$$ (6)

The evaluation function proposed in this paper, while satisfying proportionality and consistency, must also have the format of Eq (7). Since the expression is subject to degrees of freedom, it can be normalized so that the average of the values obtained is 1.

The exact definition of this mapping is presented next; for each study population, there is a vector $v,\;v\in R_{+}^{m}$, that satisfies the proportionality and consistency principles. $\Delta =\left\{X\in \frac{R_{+}^{m}}{\sum_{i=1}^{m}x_{i}=m}\right\}$, Consider the function *h*, given by Δ → *R*^*m*^,

$$h_{i}\left(x\right)=x_{i}-\frac{1}{m-1}\left(x_{i}\sum_{j\neq i}q_{ij}-\sum_{j\neq i}q_{ji}x_{j}\right)$$ (7)

where ∑_*j*≠*i*_ *q*_*ij*_ ≤ m − 1, there has Formula (8)

$$h_{i}\left(x\right)\geq x_{i}-x_{i}+\frac{1}{m-1}\sum_{j\neq i}q_{ij}x_{j}\geq 0$$ (8)

In addition, summing Eq (8) yields.


$$\sum_{i=1}^{m}h_{i}\left(x\right)=m-\frac{1}{m-1}\left(\sum_{i=1}^{m}x_{i}\sum_{j\neq i}q_{ij}-\sum_{i=1}^{m}\sum_{j\neq i}q_{ji}x_{j}\right)$$ (9)

It is important to stress that when sum $\sum_{j=1}^{m}x_{i}\sum_{j\neq i}q_{ij}=\sum_{i=1}^{m}\sum_{j\neq i}q_{ij}x_{j}$, there is $\sum_{i=1}^{m}h_{i}\left(x\right)=m$, i.e. the mapping of the function h is it itself. The function h is a continuous function and Δ is a non-empty convex set. Bloor’s theorem ([34]) ensures that there is an opportunity advantage between fixed points of the income distribution, then we have: *v* = *h*(*v*), i.e.


$$\frac{v_{j}}{v_{k}}=\frac{\sum_{j\neq k}q_{jk}}{\sum_{k\neq j}q_{kj}},i=1,2,\cdots ,m$$ (10)

### (iii) Guidelines for assessment functions

To ensure the accuracy of the above assessment functions, the principles of comparability and consistency are introduced.

#### 1. Principle of comparability

The purpose of introducing the principle of comparability is to derive an income density function. Again, considering first the simplest case, i.e., the income distribution with only two groups, for ***M*** = {***k*, *j***}, the principle of comparability makes it clear that the method we propose is to evaluate the two distributions in proportion to the corresponding probabilities of dominance and to consider in this way the "more people get more income" income The distribution. For any (***f***_***k***_, ***f***_***j***_) **∈ *p***, we have Formula (11)

$$\frac{\varphi_{k}\left(f_{k},f_{j}\right)}{\varphi_{j}\left(f_{k},f_{j}\right)}=\frac{q_{kj}}{q_{jk}}$$ (11)

The principle of comparability is the most fundamental assessment principle and provides a reasonable measure of how members of a particular group compare with members of other groups. In the case of binary group situations, the principle of comparability directly determines the outcome of the assessment, apart from the choice of units, because binary group situations that with only one degree of freedom, *φ*_*k*_ and *φ*_*j*_ multiplying simultaneously by a scalar greater than 0 does not change the result, so collapsing Eqs (11) into (12) shows that.


$$q_{jk}\varphi_{k}\left(f_{k},f_{j}\right)=q_{kj}\varphi_{j}\left(f_{k},f_{j}\right)$$ (12)

One can interpret *q*_*kj*_*φ*_*j*_ (*f*_*k*_, *f*_*j*_) as the advantage of *k* over *j*. Similarly, *q*_*jk*_*φ*_*k*_ (*f*_*k*_, *f*_*j*_) would be the advantage of *j* over *k*. The next example discusses the assessment of this property of the proportionality principle with respect to the distribution of income. The following three groups with corresponding probability densities are explored here.


$$f_{A}\left(y\right)=\left\{\begin{array}{ll} 1, & if\;y=5\\ 0, & otherwise \end{array}\right.$$ (13)

$$f_{B}\left(y\right)=\left\{\begin{array}{ll} 0.4, & if\;y=1\\ 0.6, & if\;y=3\\ 0, & otherwise \end{array}\right.$$ (14)

$$f_{C}\left(y\right)=\left\{\begin{array}{ll} 0.3, & \;if\;y=0\\ 0.2, & if\;y=0.5\\ 0.1, & if\;y=3\\ 0.4, & if\;y=4\\ 0, & otherwise \end{array}\right.$$ (15)

A two-pair study was used to compare the opportunity advantage between income distributions. It is calculated that, *q*_*AB*_ = 0.4, *q*_*BA*_ = 0.6. By standardising the values so that the mean of the assessed values equals 1, we observe that: *φ*_*A*_(*f*_*A*_, *f*_*B*_) = 0.8, *φ*_*B*_(*f*_*A*_, *f*_*B*_) = 1.2. Following the definition of the probability of dominance From the perspective of the opportunity advantage of the income distribution, it can be concluded that group B offers its group members a greater opportunity to earn a higher income compared to group A. *q*_*AC*_ = *q*_*CA*_ = 0.5, *φ*_*A*_(*f*_*A*_, *f*_*C*_) = 1; *φ*_*C*_(*f*_*A*_, *f*_*C*_) = 1, this can be interpreted as group A having the same opportunity advantage as group C. Combining the results of the comparison between A and B yields that group B offers its group members a greater probability of earning a higher income compared to group A. The results of the comparison between B and C are *q*_*BC*_ = 0.49, *q*_*CB*_ = 0.51, *φ*_*B*_(*f*_*B*_, *f*_*C*_) = 0.98, *φ*_*C*_(*f*_*B*_, *f*_*C*_) = 1.02, Let us provisionally assume that group C offers a greater opportunity advantage to its group members than group B. Clearly, this is contrary to the conclusions we have previously obtained. We therefore find that the extension of the opportunity advantage of the income distribution from binary to pluralistic groups is not feasible by relying solely on the principle of comparability, because the comparative results of the comparability principle are not transferable. How then can the opportunity advantage of the income distribution of the binary group be extended to the plural group? This requires the introduction of the principle of consistency.

#### 2. The principle of consistency

The principle of consistency is a regular requirement for the generalization of binary to multiple populations, and it usually allows the application of intuitive principles defined for two-samples to general scenarios. Here we apply this concept by relating a set of derived problems involving two distributions (each containing overall information) to any problem involving an ***m* > 2** distribution.

Generally, the most natural way of keeping track of information about all distributions while constructing into pairs of problems is to take expectations. That is, for each ${\left(f_{i}\right)}_{i=1}^{m}\in P,\;m$ two-dimensional distributions are constructed, and then the expectation is taken for each distribution. Here we define the overall advantage of group *k* as the expected advantage of *k* over all other groups, in the form of Eq (16).


$$A_{k}\left({\left(f_{i}\right)}_{i=1}^{m},\varphi \right)=\sum_{j\neq k}q_{kj}\varphi_{j}\left({\left(f_{i}\right)}_{i=1}^{m}\right)\;/\;\left(m-1\right)$$ (16)

As *m* = 2, *A*_*K*_ ((*f*_*k*_, *f*_*j*_), *φ*) = *q*_*kj*_*φ*_*j*_(*f*_*k*_, *f*_*j*_), this is the same definition as the previous one. For the dimensional probability density vector *m*, ${\left(f_{i}\right)}_{i=1}^{m}\in P$, as *m* > 2, We divide the whole group into group k and the combined group of all other groups opposed to it *M*_−*k*_, let (*f*_*k*_, *f*_−*k*_) denotes the probability density of this two-dimensional population. So, there has *f*_−*k*_ = *E*[(*f*_*j*_)_*j*≠*k*_], *E* is the mathematical expectation. *φ*_*k*_(*f*_*k*_, *f*_−*k*_), *φ*_−*k*_(*f*_*k*_, *f*_−*k*_) separate representative groups *k* valuation with consolidated group *M*-*k*, consistency introduces the principle that this merging process keeps the ratio between group evaluations in both scenarios equal to the corresponding overall advantage.

$$\frac{\varphi_{kt}\left({\left(f_{i}\right)}_{i=1}^{m}\right)}{\varphi_{kt}\left(f_{k},f_{-k}\right)}=\frac{A_{kt}\left({\left(f_{i}\right)}_{i=1}^{m},\varphi \right)}{A_{kt}\left(\left(f_{k},f_{-k}\right),\varphi \right)}$$ (17)

where *A*_*kt*_ refers to the overall expected advantage of group *k* in period *t*.

This overall expected advantage is calculated using Eq (16), the process of taking expectations for the expected advantage, while (m-1) represents the degrees of freedom for the m groups. To facilitate the reader’s understanding, a few additional notes on Eq (17) are added here. First, the numerator of Eq (17) represents the overall advantage of group k over time, which can be interpreted as the absolute advantage of group k over time; second, its denominator represents the expected advantage of all groups except group k; third, Eq (17) as a whole represents the ratio of group k’s own absolute advantage over time to the expected advantage of all the remaining groups, which can be used to reflect the change in group k’s ’s relative chance advantage, so the consistency principle can be interpreted as the ratio of its overall advantage in these two cases.

The results show that the value associated with the evaluation formula derived from the principles of comparability and consistency is proportional to its overall dominance of the income distribution and inversely proportional to the average probability of dominance of some other distribution. In other words, the process of generalizing the proportionality principle shown in Eq (17) is equivalent to the process of taking expectations.

Based on the principles of proportionality and consistency, the three income distributions mentioned above are evaluated and standardized so that their mean value is 1. The calculation gives: *φ*_*A*_(*f*_*A*_, *f*_*B*_, *f*_*C*_) = 0.868, *φ*_*B*_(*f*_*A*_, *f*_*B*_, *f*_*C*_) = 1.118, *φ*_*C*_(*f*_*A*_, *f*_*B*_, *f*_*C*_) = 1.014.

It is important to clarify that the opportunity advantage, a criterion for assessing income distribution based on the likelihood of obtaining a higher income, captures the relative opportunities that the income distribution of different groups offers to their group members from a ’curtain of ignorance’ (Wei C., [35])—a perspective that assumes absolute fairness in order to emphasize justice—and is a relative probability, not an absolute fact.

## IV. Measurement of the process of achieving common prosperity based on the advantage of opportunities

In this paper, the study participants are grouped by region, education level and gender, and the opportunity advantage approach is used to compare their opportunity differences in income distribution and thus measure the extent to which shared prosperity is achieved.

### (i) Description of data

This paper uses CFPS data from 2012–2020 in China, matching individuals’ highest education level, gender, age, current residence, urban/rural and total annual personal income data, leaving 8590 available data after excluding missing data. The data are also divided into four groups: rural/urban(urban/rural), gender (male/female), education (doctoral/master’s/bachelor’s/college/high school/junior high school/primary school/illiterate or semi-literate) and intergenerational (post-50s/post-60s/post-70s/post-80s/post-90s), to investigate the differences between the income levels of each group and their education levels, urban/rural, gender and educational attainment. Here’s the URL http://www.isss.pku.edu.cn/cfps/.

**Fig 1:**
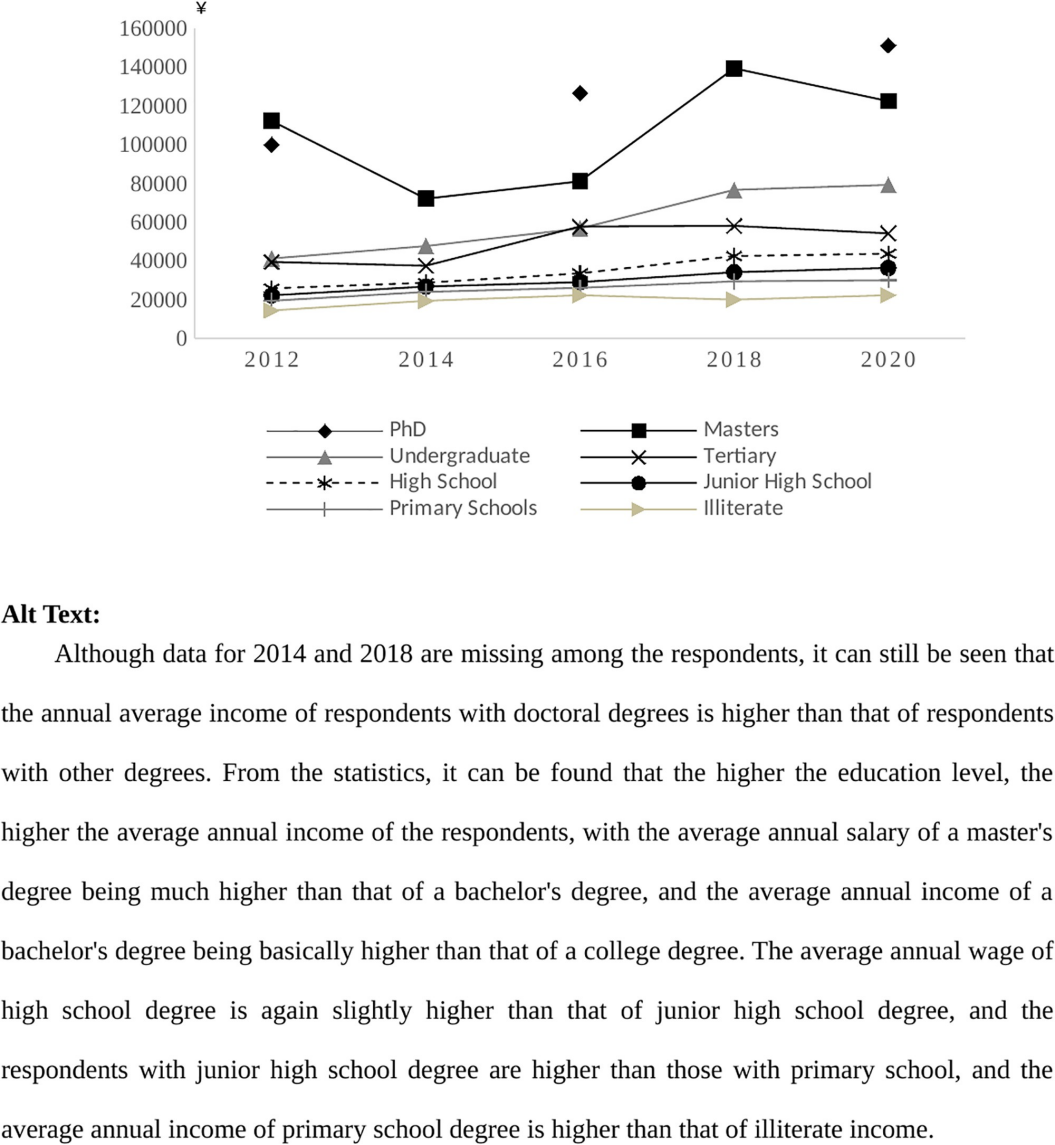
Average annual income of individuals in different education groups, 2012–2020.

Fig 1 shows the average annual personal income for different education groups from 2012 to 2020. Among them, the survey data in 2014 and 2018 were processed without the doctoral education sample, resulting in a broken distribution of the line graph for the doctoral group. As shown in Fig 1, the higher educated groups have higher income levels, as evidenced by the fact that the doctoral group earns more than the master’s group, the master’s group is significantly higher than the bachelor’s and other qualifications, while the high school and below groups are at a significant disadvantage in terms of income levels.

From Fig 1, the higher the education level, the higher the average annual income earned, and conversely, the lower the education level, the lower the average annual income earned. Looking at the urban group, the income of the highly educated group is generally higher than that of the other educated groups, and the income of those with a bachelor’s degree or less tends to increase year by year. The results of this paper are presented in the form of a table, which is attached to this article. The results show that the rural group also exhibits the same characteristics, as do the gender groups. Men’s tertiary qualifications in 2016 were higher than the earnings of those with undergraduate and other qualifications, due to insufficient sample size. As with the academic groups, the average annual income of the urban and rural groups is proportional to their academic qualifications, with the higher the academic qualification, the higher the income earned, and it should be noted that there are no PhDs in the rural group, either because they were initially based in rural areas, but moved to towns for work and other reasons after obtaining their PhDs.

The average annual income of the female master’s group in 2020 was higher than that of the male master’s group, perhaps due to the gradual increase in the proportion of women in the highly educated group. In addition, except for the doctoral degree, the urban group corresponds to a higher average annual income if the education level is the same. In terms of age groups, the higher the education, the higher the income for all groups except the post-60s. The post-60s group with a bachelor’s degree received a higher income than the master’s group, and in addition, all groups showed the characteristic that the higher the education, the higher the income. The post-70s master’s group earned the highest income among all age groups. This result is not difficult to understand, as the post-70s are more senior and influential among their peers, and are more experienced in their field of work, so they achieve higher income. Combined with the post-80s and post-90s, PhD graduates earn between ¥140,000 and ¥160,000 per year, which is in line with popular perception.

In summary, compared to women, men’s in-group earnings are higher than women’s in all groups, except for female PhDs in the gender group whose annual earnings are higher than men’s; urban earnings are higher than rural earnings between the same groups; the post-70s group with higher education has a clear advantage, followed by the post-90s, and the high school and lower education groups with lower average annual earnings are all at a disadvantage in terms of obtaining higher levels of income.

### (ii) Opportunity advantage measurement

To measure the opportunity advantage of each group by region, we first grouped the data according to gender, education level, and the region in which the respondents lived. It should be noted here that, to reduce errors, we have excluded respondents under the age of 18 and those who are not yet involved in social production activities (these members are mainly students) before grouping them. The opportunity advantage was then measured and compared across regions and groups in accordance with the theory of opportunity advantage based on income distribution presented in the second part of the paper.

As the gender grouping is two-dimensional, the 2-dimensional theory of opportunity advantage was chosen. As both the education level and the region of the respondents were divided into 4 groups, the theory of dominance probability under the 4-dimensional opportunity advantage was chosen. The measurement process is strictly in accordance with the opportunity advantage theory presented in the second part of this paper, and will not be repeated here, as the process of measuring the opportunity advantage for the gender group in 2020 is used as an example, while the rest of the results are given directly.

The density function for the gender group is first given:

$$f_{1}\left(y\right)=\left\{\begin{array}{ll} 0.41, & if\;\;y=3.8\\ 0.59, & if\;\;y=0.52\\ 0, & otherwise \end{array}\right.$$ (18)

$$q_{mf}=\int_{0}^{+\infty }f_{f}\left(y\right)\int_{0}^{y}f_{m}\left(t\right)dtdy$$ (19)

Taking (18) into (19) gives *q*_*mf*_ = 0.67. and *q*_*fm*_ = 0.33, Δ = 0.67–0.33 = 0.34. It follows that men have a greater chance of earning a higher income compared to women. The results of the measurements are shown in Table 1.

**Table 1:** Results of the 2020 opportunity advantage measure.

Study Subjects	variables	Probability	Group Ranking	Comparator	Level of variation
Male	*q*_*mf*_	0.67	1	(M, Fm)	0.34
Female	*q*_*fm*_	0.33	2	(T, R)	0.13
Total	1			(P, M)	0.05
Town	*q*_*cn*_	0.58	1	(P, U)	0.13
Rural	*q*_*nc*_	0.45	2	(P, T)	0.18
Total	1			(M, U)	0.08
PhD	*φ*_*A*_(*f*_*A*_, *f*_*B*_, *f*_⋯_, *f*_*H*_)	0.28	1	(M, T)	0.13
Masters	*φ*_*B*_(*f*_*A*_, *f*_*B*_, *f*_*C*_, *f*_*D*_)	0.23	2	(M, H)	0.15
Undergraduate	*φ*_*C*_(*f*_*A*_, *f*_*B*_, *f*_⋯_, *f*_*H*_)	0.15	3	(U, T)	0.05
Tertiary	*φ*_*D*_(*f*_*A*_, *f*_*B*_, *f*_⋯_, *f*_*H*_)	0.10	4	(U, H)	0.07
High School	*φ*_*E*_(*f*_*A*_, *f*_*B*_, *f*_⋯_, *f*_*H*_)	0.08	5	(U, J)	0.08
Junior	*φ*_*F*_(*f*_*A*_, *f*_*B*_, *f*_⋯_, *f*_*H*_)	0.07	6	(T, H)	0.02
Primary	*φ*_*G*_(*f*_*A*_, *f*_*B*_, *f*_⋯_, *f*_*H*_)	0.05	7	(T, J)	0.03
Illiterate	*φ*_*H*_(*f*_*A*_, *f*_*B*_, *f*_⋯_, *f*_*H*_)	0.04	8	(T, P)	0.05
Total	1			(H, J)	0.01
East	*φ*_*L*_(*f*_*L*_, *f*_*M*_, *f*_*W*_)	0.49	1	(H, P)	0.03
Medium	*φ*_*M*_(*f*_*L*_, *f*_*M*_, *f*_*W*_)	0.31	2	(E, M)	0.18
West	*φ*_*W*_(*f*_*L*_, *f*_*M*_, *f*_*W*_)	0.20	3	(E, W)	0.29
Total	1			(M, W)	0.11

As can be seen from Table 1, the probability of dominance for men is 0.67, which is greater than the probability of dominance for women, so men have a greater probability of earning a higher income than women. This indicates that there is still a degree of gender discrimination in our social development process. The difference between urban and rural areas persists, with urban residents having a higher probability of earning a higher income than rural residents, and the difference between the two reaching 0.13, indicating that the process of urban-rural integration still needs to be continued.

In terms of education, the educational attainment of women was significantly higher than that of men. Respondents with postgraduate and higher education have a higher opportunity advantage than those with undergraduate and tertiary education, with a difference of 0.21. Undergraduate and tertiary education is in turn higher than high school education, with a difference of 0.19. Similarly, high school education has a higher opportunity advantage than respondents with lower secondary education and below, but the gap between high school education, lower secondary education and below is significantly smaller compared to the other adjacent spacing, at 0.08.

Looking at the division of the three major economic regions, residents of the eastern region have a greater probability of earning a higher income compared to residents of the other two regions, followed by the central region and finally the western region. This indicates that, among the three major economic regions in China, residents of the eastern region have a greater advantage in terms of the probability of obtaining a higher income, and the difference in opportunity advantage between the three is obvious. To a certain extent, this reveals that China should pay attention to a high degree of balance among regions in terms of sharing opportunities and fruits on the road to achieving common prosperity for all people. The differences in opportunities between regions in the process of achieving common prosperity can be attributed to resource endowments. The eastern region, mainly the coastal region, is more open than the inland region, with a superior geographical location and a relatively developed economy, thus creating an imbalance between regions, and in order to further weaken the imbalance between regions, it is necessary to develop regional characteristic economies according to local conditions in order to create a situation of common prosperity for all regions in a relatively short period of time.

In terms of the level of disparity, there are significant differences in the level of opportunity disparity by gender, educational attainment and the three main economic regions. The process of calculating the level of disparity is relatively simple, i.e., the difference in the probability of dominance between the different study subjects, and it is this that is used as a measure of the process of promoting common prosperity in this paper. This level of opportunity differential should be different at different stages of development and should converge to zero as the process of achieving common prosperity in China continues to develop, while the decomposition and definition of the process of achieving common prosperity will be given in a separate article.

In the gender subgroup, the difference in probability between males and females in obtaining a higher level of income is 0.34. Although this value only represents the difference between males and females on average among the respondents to this survey, it still indicates a degree of difference between gender and income level, suggesting that females are discriminated against in obtaining a higher level of income.

The level of difference in opportunity advantage between groups also shows different characteristics in terms of educational attainment groupings. Table 1 presents the opportunity advantages corresponding to each group with different levels of education and lists the levels of difference between groups with relatively similar levels of education, such as PhD and Master’s, PhD and Bachelor’s, and PhD and specialist. The difference between doctoral and bachelor’s degrees is larger, with the former having an opportunity advantage of 0.23 and the latter only 0.15, with a difference of 0.08. The difference between bachelor’s and specialist degrees is also significant, at 0.05. The difference between high school, junior high school, primary school, and illiterate people is smaller. The difference in chance between high school, junior high school, primary school, and illiterate is smaller. This shows that there is a significant difference between high and low education in obtaining higher income levels, and that respondents with high education above master’s degree possess a greater opportunity advantage than those with high school, junior high school and below. We found that there are two main features of the difference in opportunity advantage between different levels of education: firstly, those with a bachelor’s degree or above have a greater opportunity advantage than those with high school, junior high school or below; secondly, the level of opportunity difference is distributed in a stepwise manner, i.e., the greater the difference in education, the greater the opportunity difference in gaining higher income.

Among the three major economic regional groupings, the difference in opportunity advantage between the eastern and western regions is the most pronounced, with a difference value of 0.29, followed by the east-central difference, with a difference value of 0.18. The east-central difference is significantly lower than the two, by 0.11. The value of the east-west difference is significantly lower than the east-central difference and the east-west difference, as both the east and west regions are less developed, so the opportunity difference between them is 0.11, but still shows that the central region is higher than the western region.

The above results are consistent with the fact that, in general, the higher the level of education, the higher the level of income, which can be interpreted as a mechanism to compensate for the cost of higher education (Suo N., [36]). Based on the inherent objective requirement of social equity, the more capable party will bear more costs, while the relatively less capable individuals, the low-income groups, will bear less education costs accordingly, and both individuals and groups gain different degrees of benefits from higher education.

As can be seen from Table 2, all groups show that the higher the level of education, the greater the probability of having a higher income, except for the male group where a master’s degree has a greater opportunity advantage over a doctorate. In terms of gender, the opportunity advantage was significantly higher for males compared to females within the group. There is also a greater probability of higher earnings in urban compared to rural areas between groups, whereas this trend is not evident between groups.

**Table 2:** Results of the opportunity advantage measure 2012–2020.

Average						Age					
Edit	2012	2014	2016	2018	2020	Age	2012	2014	2016	2018	2020
Ph	0.36	0.38	0.34	0.30	0.28	50s	0.11	0.12	0.11	0.12	0.11
M	0.23	0.22	0.22	0.20	0.23	60s	0.14	0.17	0.14	0.17	0.18
U	0.15	0.13	0.13	0.14	0.15	70s	0.32	0.21	0.32	0.21	0.21
T	0.14	0.11	0.10	0.11	0.10	80s	0.25	0.29	0.25	0.29	0.27
H	0.05	0.05	0.07	0.09	0.08	90s	0.18	0.21	0.18	0.21	0.23
J	0.04	0.04	0.06	0.07	0.07						
Pr	0.02	0.03	0.05	0.05	0.05						
I	0.01	0.04	0.03	0.04	0.04						
Male						Female					
Edit	2012	2014	2016	2018	2020	Edit	2012	2014	2016	2018	2020
Ph	0.31	0.35	0.28	0.28	0.23	Ph	0.37	0.37	0.38	0.37	0.87
M	0.22	0.22	0.20	0.23	0.25	M	0.24	0.22	0.23	0.24	0.04
U	0.15	0.12	0.15	0.15	0.16	U	0.13	0.14	0.13	0.13	0.03
T	0.13	0.11	0.13	0.11	0.11	T	0.10	0.12	0.09	0.10	0.02
H	0.08	0.07	0.09	0.08	0.08	H	0.07	0.07	0.07	0.07	0.01
J	0.07	0.06	0.07	0.07	0.07	J	0.05	0.04	0.05	0.05	0.01
Pr	0.03	0.04	0.05	0.05	0.06	Pr	0.03	0.03	0.03	0.03	0.01
I	0.01	0.03	0.03	0.03	0.04	I	0.01	0.01	0.02	0.01	0.04
Urban						County					
Edit	2012	2014	2016	2018	2020	Edit	2012	2014	2016	2018	2020
Ph	0.34	0.35	0.34	0.31	0.28	Ph	----	----	----	----	----
M	0.22	0.22	0.22	0.22	0.22	M	0.40	0.38	0.40	0.38	0.37
U	0.12	0.13	0.12	0.13	0.15	U	0.19	0.15	0.16	0.15	0.17
T	0.09	0.10	0.09	0.10	0.10	T	0.16	0.12	0.12	0.12	0.12
H	0.08	0.08	0.08	0.08	0.08	H	0.09	0.11	0.09	0.11	0.11
J	0.07	0.07	0.07	0.07	0.07	J	0.07	0.10	0.10	0.10	0.10
Pr	0.05	0.03	0.06	0.05	0.06	Pr	0.05	0.08	0.07	0.08	0.08
I	0.03	0.02	0.02	0.04	0.04	I	0.04	0.06	0.06	0.06	0.06

Note: All letters in the table are the first letter or the first 1 or 2 letters of the different qualifications in Table 1 and have the same meaning as in Table 1.

The academic group, the urban group, the rural group, the male group, and the female group all show a higher probability of domination with higher education, with the probability of domination being higher in the urban than in the rural group for both the male and female groups, and the probability of domination being higher in the urban than in the rural group for both the male and rural groups. From 2018 to 2020, the difference in opportunity advantage gradually decreases. For example, if the probability of dominance for the academic group (PhD) in 2018 is 0.30 and the probability of dominance for the master’s group is 0.20, with a difference value of 0.1, while the probability of dominance for the academic group in 2020 is 0.28 for the PhD and 0.23 for the master’s group, the difference value is 0.05, which changes from 0.1 to 0.05, and the opportunity difference value narrows by 0.05, indicating that the common wealth in 2020 level has made significant progress compared to 2018.

### (iii) Measurement of the process of achieving common prosperity

Above measures the opportunity advantage of each group based on income distribution. Since the promotion of equity of opportunity is highly compatible with the promotion of common prosperity, the difference in opportunity advantage between groups can be used to characterize the process of achieving common prosperity. To this end, the difference in opportunity advantage between and within groups is measured separately for the period 2012–2020 to gauge the process of achieving shared prosperity.

Table 3 shows the opportunity difference between groups using the years 2012–2020, a positive value indicates a greater advantage for the former, while its negative value indicates a greater opportunity advantage for the latter, e.g. in 2012 the opportunity difference between the post-50s and the post-60s is 0.01, indicating that the post-50s have a greater probability of earning a higher income than the post-60s in 2012, while by 2014 the opportunity The difference of -0.01 indicates that the post-60s have surpassed the post-50s in having a greater opportunity advantage. This shows that men have an opportunity advantage over women, but that this difference in opportunity advantage is diminishing year on year, and that the opportunity difference between urban and rural areas shows the same characteristics. The opportunity advantage within each age group is greatest for the post-70s, followed by the post-80s, followed by the post-90s, and then the post-60s and post-50s respectively. the post-70s are more likely to have a higher income due to their seniority, the post-80s and post-90s also have an advantage as the backbone of society, and the post-50s and post-60s do not have a sufficient advantage to have a higher income due to their age and other reasons. The larger value of 0.44 for the opportunity difference between men and women in 2016 should be due to data bias. Overall, the differences between groups, although fluctuating, are all around 0 and show a tendency to gradually move closer to 0, indicating that opportunities are becoming more equitable, and the degree of common prosperity realization has been effectively developed.

**Table 3:** Differences in opportunities between groups, 2012–2020.

Comparator	2012	2014	2016	2018	2020
(F, Fm)	0.15	0.14	0.44	0.00	0.34
(Ur, Ct)	0.31	0.13	0.12	0.08	0.13
(50s, 60s)	0.01	-0.10	-0.07	-0.13	-0.07
(50s, 70s)	-0.11	-0.11	-0.12	-0.15	-0.17
(50s, 80s)	-0.06	-0.15	-0.20	-0.15	-0.12
(50s, 90s)	0.03	-0.12	-0.19	-0.12	-0.09
(60s, 70s)	-0.12	-0.01	-0.05	-0.02	-0.10
(60s, 80s)	-0.07	-0.05	-0.13	-0.02	-0.05
(60s, 90s)	0.02	-0.02	-0.12	0.01	-0.02
(70s, 80s)	0.05	-0.04	-0.08	0.00	0.05
(70s, 90s)	0.14	-0.01	-0.07	0.03	0.08
(80s, 90s)	0.10	0.03	0.01	0.03	0.03

Note: All letters in the table are the first letter or the first 1 or 2 letters of the different qualifications in Table 1 and have the same meaning as in Table 1.

Table 4 measures the difference in the opportunity advantage of different education levels within different age groups from 2012 to 2020 and finds that the larger the gap in education levels, the larger the difference in opportunity advantage, and the smaller the gap in education levels, the smaller the difference in opportunity. Those with a bachelor’s degree or higher education have a greater opportunity advantage, but this difference is gradually weakening, and those with high school or lower education are at a disadvantage in different age groups, and the opportunity difference is close to 0. Education shows that education equity plays an important role in promoting the degree of opportunity equity and common prosperity. From 2012 to 2020, the opportunity gap between groups gradually decreases, for example, the opportunity gap between post-60s doctoral and master’s degree groups decreases from 0.15 in 2012 to 0.07 in 2020, while the opportunity gap between doctoral and bachelor’s degree groups decreases from 0.24 to 0.17, indicating that the degree of achievement of common prosperity is firmly in progress.

**Table 4:** Within-group opportunity differences by age group, 2012–2020.

Edit	2012	2014	2016	2018	2020	2012	2014	2016	2018	2020
	60s					70s				
(Ph, M)	0.15	0.14	0.12	0.09	0.07	0.11	0.10	0.08	0.07	0.05
(Ph, U)	0.24	0.32	0.31	0.21	0.17	0.11	0.20	0.19	0.18	0.16
(Ph, T)	0.19	0.25	0.19	0.15	0.20	0.11	0.17	0.22	0.15	0.11
(Ph, H)	0.22	0.12	0.17	0.08	0.10	0.16	0.15	0.16	0.10	0.07
(Ph, J)	0.29	0.12	0.12	0.07	0.09	0.17	0.11	0.11	0.09	0.06
(Ph, Pr)	0.37	0.11	0.12	0.06	0.07	0.18	0.09	0.10	0.07	0.05
(Ph, I)	0.37	0.11	0.12	0.06	0.07	0.19	0.08	0.09	0.06	0.04
(M, U)	0.21	0.32	0.31	0.19	0.10	0.23	0.04	0.32	0.19	0.35
(M, T)	0.24	0.25	0.19	0.24	0.03	0.22	0.03	0.22	0.21	0.40
(M, H)	0.21	0.12	0.17	0.11	0.07	0.28	0.08	0.16	0.25	0.44
(M, J)	0.23	0.12	0.12	0.32	0.08	0.28	0.09	0.11	0.27	0.45
(M, Pr)	0.26	0.21	0.12	0.18	0.10	0.30	0.11	0.11	0.28	0.46
(M, I)	0.16	0.11	0.12	0.33	0.10	0.30	0.12	0.10	0.30	0.46
(U, T)	0.05	0.07	0.12	0.05	0.07	0.01	0.07	0.09	0.02	0.06
(U, H)	0.12	0.20	0.14	0.13	0.16	0.05	0.12	0.10	0.06	0.09
(U, J)	0.15	0.20	0.19	0.13	0.18	0.05	0.13	0.16	0.08	0.10
(U, Pr)	0.17	0.21	0.19	0.14	0.20	0.07	0.15	0.21	0.09	0.11
(U, I)	0.17	0.21	0.19	0.14	0.20	0.08	0.16	0.22	0.11	0.12
(T, H)	0.07	0.12	0.03	0.07	0.09	0.06	0.05	0.23	0.04	0.04
(T, J)	0.09	0.13	0.07	0.08	0.11	0.06	0.06	0.06	0.06	0.04
(T, Pr)	0.11	0.14	0.08	0.09	0.13	0.08	0.08	0.07	0.07	0.06
(T, I)	0.11	0.14	0.08	0.09	0.13	0.08	0.08	0.09	0.09	0.06
(H, J)	0.02	0.00	0.04	0.01	0.02	0.00	0.00	0.02	0.02	0.01
(H, Pr)	0.05	0.01	0.05	0.02	0.03	0.02	0.02	0.03	0.03	0.02
(H, I)	0.05	0.01	0.05	0.02	0.03	0.03	0.03	0.04	0.04	0.03
(J, Pr)	0.02	0.01	0.01	0.01	0.02	0.01	0.02	0.01	0.01	0.01
(J, I)	0.02	0.01	0.01	0.01	0.02	0.02	0.03	0.02	0.03	0.02
(Pr, I)	0.00	0.00	0.00	0.00	0.00	0.01	0.01	0.01	0.01	0.01
	80s					90s				
(Ph, M)	0.13	0.11	0.09	0.09	0.07	0.12	0.13	0.14	0.09	0.08
(Ph, U)	0.15	0.18	0.06	0.17	0.12	0.16	0.15	0.14	0.14	0.13
(Ph, T)	0.18	0.17	0.08	0.15	0.17	0.21	0.16	0.17	0.15	0.14
(Ph, H)	0.20	0.19	0.18	0.16	0.16	0.21	0.12	0.23	0.12	0.20
(Ph, J)	0.23	0.20	0.19	0.19	0.20	0.25	0.21	0.19	0.20	0.19
(Ph, Pr)	0.25	0.23	0.21	0.17	0.16	0.27	0.21	0.22	0.19	0.21
(Ph, I)	0.25	0.20	0.22	0.19	0.18	0.28	0.24	0.25	0.16	0.18
(M, U)	0.30	0.13	0.05	0.19	0.06	0.26	0.10	0.04	0.10	0.09
(M, T)	0.34	0.16	0.08	0.21	0.10	0.21	0.13	0.02	0.16	0.13
(M, H)	0.34	0.20	0.12	0.25	0.12	0.21	0.15	0.09	0.17	0.12
(M, J)	0.36	0.21	0.14	0.27	0.14	0.15	0.16	0.09	0.20	0.14
(M, Pr)	0.36	0.22	0.15	0.28	0.14	0.10	0.16	0.10	0.20	0.14
(M, I)	0.39	0.23	0.15	0.30	0.17	0.08	0.18	0.10	0.24	0.17
(U, T)	0.04	0.04	0.02	0.02	0.04	0.06	0.04	0.02	0.06	0.04
(U, H)	0.05	0.07	0.07	0.06	0.06	0.06	0.05	0.04	0.07	0.04
(U, J)	0.07	0.08	0.09	0.08	0.08	0.12	0.06	0.05	0.10	0.05
(U, Pr)	0.07	0.09	0.10	0.09	0.09	0.16	0.06	0.06	0.11	0.05
(U, I)	0.09	0.11	0.10	0.11	0.11	0.18	0.08	0.06	0.14	0.09
(T, H)	0.01	0.03	0.04	0.04	0.02	0.00	0.01	0.07	0.01	0.00
(T, J)	0.03	0.05	0.07	0.06	0.04	0.06	0.02	0.07	0.04	0.01
(T, Pr)	0.03	0.05	0.07	0.07	0.04	0.11	0.03	0.08	0.05	0.01
(T, I)	0.05	0.07	0.08	0.09	0.07	0.13	0.05	0.08	0.08	0.05
(H, J)	0.02	0.01	0.03	0.02	0.02	0.06	0.01	0.01	0.02	0.02
(H, Pr)	0.02	0.02	0.03	0.03	0.03	0.11	0.01	0.02	0.03	0.01
(H, I)	0.05	0.04	0.04	0.04	0.05	0.13	0.04	0.02	0.07	0.05
(J, Pr)	0.00	0.01	0.01	0.01	0.01	0.05	0.00	0.01	0.01	0.00
(J, I)	0.03	0.02	0.01	0.03	0.03	0.07	0.02	0.01	0.04	0.03
(Pr, I)	0.03	0.02	0.00	0.01	0.02	0.02	0.02	0.00	0.03	0.04

Note: All letters in the table are the first letter or the first 1 or 2 letters of the different qualifications in Table 1 and have the same meaning as in Table 1.

Tables 3 and 4 measure the opportunity differences between and within groups respectively, as a way of characterizing the degree of achievement of common prosperity and conclude that the degree of achievement of common prosperity in China has steadily increased both between and within groups from 2012 to 2020. To test the reliability of this finding, a significance test was conducted using the multiple comparison method (LSD) and the results are shown in Table 5.

**Table 5:** LSD method significance test.

year	2020	2018	2016	2014
2012	***	**	*	**
2014	*	*	*	
2016	*	*		
2018	*			

Note:

*** indicates significant at the 0.01 level,

** indicates significant at the 0.05 level and

* indicates significant at the 0.1 level.

As can be seen from Table 5, the differences are significant in all years, with the value of opportunity differences between 2012 and 2020 significant at the 0.01 level. Combined with Tables 3 and 4, the opportunity differences in access to higher income for each group from 2012 to 2020 show different magnitudes of decline, indicating that the degree of common prosperity realization has been effectively increased from 2012 to 2020, further validating the conclusions of this paper.

## V. Discussion and conclusion

### (i) Discussion

With the convening of the Fifth Plenary Session of the Nineteenth Central Committee of China 2020 and the comprehensive victory in China’s battle against poverty, a considerable amount of research has been carried out by academics around common wealth. It mainly covers the connotation and measurement of common wealth and the path to realization. This paper focuses on the measurement of common wealth, so it focuses on the relevant literature on the measurement of common wealth. Based on the concept of sharing the fruits of development, Chao and Ren [6] constructed an evaluation index system of common prosperity from the dimensions of income and wealth, development capacity and people’s well-being, and put forward the idea of weighting and index synthesis; Li [2] put forward a phased target in the three dimensions of income, property and basic public services from the perspective of the level of development and sharing; and Wan and Chen [14] take the per capita national income, the Gini coefficient of disposable income, etc. to reflect the level of development and sharing, and construct the evaluation index system of common wealth from the development and sharing dimensions. There are numerous similar measurement methods, which are relatively widely used due to their simplicity. Meanwhile, due to the different indicators and weighting methods chosen by scholars, the results of the measurements are different and cannot be directly compared. However, the above literature shows that the construction of common wealth in China has achieved some success. In addition to the similarity of the conclusions, it is not difficult to find that the above literature also focuses on the key words "sharing" and "affluence". Unlike the above studies, this paper does not adopt the method of constructing an indicator system to measure the common wealth, but measures the advancement of the common wealth from the perspective of "equal economic opportunities" based on the comparative method of opportunity advantage of income distribution, which is actually not detached from the words "sharing" and "affluence". In fact, it has not been separated from "sharing" and "affluence". At the same time, the findings of this paper are like those of existing literature, which confirms the reliability of this paper’s findings. In addition, compared with other literature, this paper analyses from a micro perspective, while most of the existing literature uses macro data to measure it, which is also regarded as a supplement to the gap in the existing literature.

Using the latest data from CHIP 2018, Shi et al. [5] provide a systematic analysis of how the lack of opportunities affects income jumps for low-income groups from a doctrinal perspective by introducing and expanding the analytical framework of inequality of opportunity. finds that whether an individual is caught in a low-income trap depends largely on opportunity factors that are difficult to change once the individual is born. Based on parameter estimation and machine learning methods, the coefficient of opportunity inequality associated with individual low-income status is found to range from 0.310 (parameter estimation) to 0.336 (machine learning), which means that more than 30% of the low-income status is associated with differences in opportunity factors. The difference between this measure and this paper is not significant, which confirms the reliability of the results of this paper.

In addition, there are some limitations in the measurement methodology of this study; this paper also focuses on the economic opportunities and income distribution of residents, etc., but at the same time ignores other factors that may contribute to the realization of common wealth in China, such as digital green innovations, etc. (Yin and Zhao,[37]; Dong et al.[38]). It also does not consider the regional differences and spatial correlation that may exist in the process of realizing common wealth (Zhao et al. [39]). We will try to address these issues in our future research.

### (ii) Conclusion

The methodology proposed in this paper for comparing the advantages of opportunities based on income distribution is a good measure of the process of realizing common prosperity in China, focusing on "prosperity" as well as "equal opportunities", although we still emphasize economic opportunities. Compared with the rest of the studies that measure common wealth by way of constructing an indicator system, the research methodology of this paper satisfies two features, the principle of horizontal comparability and vertical consistency, i.e., different groups of researchers can be compared within the same research period, and the same research object can be compared across different periods of time, in order to measure the advancement of common wealth. The study found that since 2012–2020, the difference in the opportunity advantage of income distribution has been gradually reduced, indicating that the process of realizing common wealth in China is being effectively promoted. Firstly, there is a great deal of heterogeneity in the process of realizing common prosperity in different regions, groups, and areas of China. From the perspective of different regions, in descending order of opportunity advantages, the eastern, central, western, and north-eastern regions are. That is, residents of the East have a greater probability of earning higher incomes, while residents of the Northeast have a significantly lower probability of earning higher incomes than the other three regions. Second, among the different subgroups of educational status, the opportunity advantage of respondents with graduate and higher education is significantly higher than that of respondents with undergraduate and college education, and the opportunity advantage of respondents with undergraduate and college education is higher than that of respondents with high school education, and respondents with junior high school education and below do not have any advantage in terms of the opportunity advantage, i.e., the group of people with junior high school education and below has the probability of obtaining a higher income is not significant. This can be summarized as the higher the level of education, the greater the chances of earning higher incomes, but the qualification dividend is depreciating, i.e. the probability of relying on qualifications for higher incomes is lower than in the past. It shows that social development has become more diversified and there is no longer a single way for people to obtain income. This points to a direction for us to realize the common wealth of all the people, that is, in the future, society should pay more attention to education, raise the average level of education of the whole population, and provide certain policy assistance to members of society with a lower level of education. Thirdly, there are large group differences. Men have a higher opportunity advantage than women, and urban residents have a higher opportunity advantage than rural residents. This shows that, on the road to realizing the common prosperity of all people, breaking down gender differences and urban-rural differences remains a top priority.

### (iii) Policy insights

Based on the findings of this paper, the following are the main policy implications.

First, the income gap should be continuously narrowed. Narrowing the income gap is the core element in promoting common prosperity for all people, and the income gap between groups, between urban and rural areas and between regions should be our focus of attention. Allowing a certain amount of income disparity may burst out more market vitality, but the income disparity should be kept within a reasonable range to enable the country to enjoy long-term stability and the people to live and work in peace and happiness.

Secondly, transfer payments should be appropriately increased to protect the income of low-income earners. On the path of promoting common prosperity for all people, our society should be getting better and better, and all members of society should effectively feel the dividends of social development and progress. Therefore, we can appropriately increase transfer payments to protect the income of the lowest-income earners and meet their basic needs of living.

Thirdly, we should increase investment in education and adhere to the college entrance examination system. Increase investment in education, which is the foundation of a strong nation, ensure absolute equity in education opportunities, optimize the allocation of educational resources, and ensure that young people have access to education and learning. The only way to promote common prosperity for all people is to take a step-by-step approach and advance in all directions. An education compensation mechanism should be set up to break down inter-generational barriers and strive to give more people a greater chance to earn a higher income.

Fourthly, for some regions and groups that are lagging behind in economic development, an opportunity compensation mechanism should be proposed to provide them with some development opportunities as appropriate, so that they can quickly integrate into the general environment of the rapid development of the country as a whole and not hold back the achievement of common prosperity for all people, thus realizing the great situation of common prosperity for all people and common sharing.

### (iv) Limitations

In fact, this paper still suffers from several limitations. Firstly, this paper also focuses on considering the income and economic opportunities of residents, without considering other dimensions such as public services, which may result in insufficient explanation of common wealth; secondly, compared with other literatures, this paper is from a micro perspective, considering the differences between groups, and does not take into account the differences in opportunities within the group that may be caused by the lack of individual efforts; thirdly, the measurement method of common wealth has not yet formed a complete theoretical framework, and this paper only starts from a micro perspective and adds the consideration of micro individuals and local groups, but does not build up the "individual-group". Thirdly, there is no complete theoretical framework for the measurement of common wealth, this paper only starts from the micro perspective, adds the consideration of micro individuals and local groups, but does not build up the measurement method of "individual-group-whole", we will try to solve the above problems in our future research. We will try to solve the above problems in our future research.

## Supporting information

S1 Appendix (DOCX)
