# Supplementary material for: Analyzing the process of achieving common wealth for different groups in China based on the opportunity advantage perspective of income distribution
Source: PLoS One. 2024 May 9;19(5):e0302876. doi: 10.1371/journal.pone.0302876 (PMC11081256; doi:10.1371/journal.pone.0302876)
Supplement: S1 Appendix — (DOCX) [file pone.0302876.s001.docx]

**Appendix**

**Appendix Table 1 Literature Collation**

| **cited location** | **authors** | **Perspectives or methods** | **Differences and linkages** |
| --- | --- | --- | --- |
| Part 3.2 | **Bloor D.** (1991) | Knowledge and Social Imagery | Knowledge is the basis for the grouping of studies in this paper. |
| Review of literature | **Chao X.J., and Ren B.P.** (2022) | indexation technique | Both use index construction to measure the degree of realization of common wealth. |
| Review of literature | **Li J.C., and Yu W.** (2022) | indexation technique |  |
| Review of literature | **Lv G.M., and Chen X.Y.** (2022) | indexation technique |  |
| Literature review and introduction | **Liu P.L., Qian T., and Huang X.H., et al.** (2021) | Indicators such as Gini coefficient, Tel index | Both use indicators such as the Gini coefficient and the Thiel index to measure common wealth |
| Review of literature | **Tong M.H., Chu C. C., and Li Y.** (2022) | Indicators such as Gini coefficient, Tel index |  |
| Review of literature | **Wan, H.Y., and Chen, K.P.** (2021) | Indicators such as Gini coefficient, Tel index |  |
| Review of literature | **Zhu Z.C., Liu B., and He J.** (2022) | Indicators such as Gini coefficient, Tel index |  |
| Review of literature | **Fu C.W., and Gao W.** (2022) | The meaning of common wealth | Income, especially income inequality, affects shared prosperity |
| Review of literature | **Li H.J., and Du S.** (2022) | The meaning of common wealth |  |
| Review of literature | **Chen Y.J., and Yao M.L.** (2012) | Income is closely related to shared prosperity |  |
| Review of literature | **Luo B.L., Hong W.J., and Geng P.P., et al.** (2021) | Income inequality affects shared prosperity |  |
| Review of literature | **Cheng, C., and Ke, X.W.** (2022) | Income is often used to study inequality | The importance of income in the study of inequality and shared prosperity |
| Review of literature | **Su D.W., and Ye J.J.** (2021) | Income is often used to study inequality |  |
| Review of literature | **Li C., Liu R.H., and Xu Y.J. (2019)** | Income is often used to study inequality |  |
| Review of literature | **Hou X.D., Zhu Q.L., and Wan C.F.** (2022) | The core issue of common wealth is to reduce the income gap |  |
| Review of literature | **Li S.** (2021) | The core issue of common wealth is to reduce the income gap |  |
| Review of literature | **Feng L., Liu Q., Xu S.** (2021) | Imbalance is an important issue | Where inequality is typically manifested highlights the measurement focusses of this paper |
| Review of literature | **Han W.L., and Tang X.** (2022) | The method of distribution is also critical |  |
| Review of literature | **Lai D.S.** (2021) | as above |  |
| Review of literature | **Huang Y., Ren G.Q., and Zhou Y.B.** (2019) | Opportunity advantages are usually expressed in terms of gender, urban/rural, ethnicity or income. |  |
| Review of literature | **Hou Y.B., and Ge L.Y.** (2020) | as above |  |
| Review of literature | **Li H.Y., and He Y.** (2019) | as above |  |
| Review of literature | **Yue J.L., Fang K., and Jiang Z.Y.** (2020) | as above |  |
| Review of literature | **Li S., and Zhu M.B.** (2022) | Income distribution | Together, this serves as the theoretical basis for the paper's proposal to use a comparative approach to opportunity advantage based on income distribution. |
| Literature review and introduction | **Li S.** (2021) | The goal of common wealth and the path to its realization |  |
| Part 4.2 | **Suo N.** (2020) | Relationship between income level and educational attainment |  |
| Review of literature | **Sun Y., and Zhang J**. (2022) | Two important aspects of shared prosperity are equity and efficiency |  |
| Review of literature | **Yao D.Z.** (2022) | Equal opportunities under conditions of absolute equity |  |
| Review of literature | **Mou C.W., Liu P.L.** (2020) | Rural Relative Poverty | Aspects that should be addressed in the measurement and realization of shared prosperity |
| Review of literature | **Wu Z.L., Wang L.** (2020) | Rural Relative Poverty |  |
| Review of literature | **Zhang Y.L., XU L.M.** (2020) | Rural Relative Poverty |  |
| Review of literature | **Zhou G.H., Zhang R.J., He Y.H.** (2020) | Rural Relative Poverty |  |
| Review of literature | **Carmen H., and Antonio V.** (2021) | Application of Opportunity Advantage | Although opportunity advantage is applied, only inequality is discussed, not shared prosperity |
| Part 3.2.2 | **Wei C.** (2019) | Absolute Fairness Under the Curtain of Ignorance | Introducing the concept of opportunity advantage under conditions of absolute equity |
| Review of literature | **Rawls J.** (1971) | Presentation of the theory of absolute equity | Absolute equity theory is a prerequisite for comparisons of opportunity advantages |
